# Pyroptosis and gasdermins—Emerging insights and therapeutic opportunities in metabolic dysfunction-associated steatohepatitis

**DOI:** 10.3389/fcell.2023.1218807

**Published:** 2023-08-17

**Authors:** Christian Stoess, Aleksandra Leszczynska, Lin Kui, Ariel E. Feldstein

**Affiliations:** ^1^ Department of Pediatric Gastroenterology, University of California, San Diego, San Diego, CA, United States; ^2^ Department of Surgery, TUM School of Medicine, Klinikum rechts der Isar, Technical University of Munich, Munich, Germany

**Keywords:** pyroptosis, gasdermins, liver, steatotic liver disease, steatohepatitis, MASH, MASLD

## Abstract

In recent years, there has been a rapid expansion in our understanding of regulated cell death, leading to the discovery of novel mechanisms that govern diverse cell death pathways. One recently discovered type of cell death is pyroptosis, initially identified in the 1990s as a caspase-1-dependent lytic cell death. However, further investigations have redefined pyroptosis as a regulated cell death that relies on the activation of pore-forming proteins, particularly the gasdermin family. Among the key regulators of pyroptosis is the inflammasome sensor NOD-like receptor 3 (NLRP3), a critical innate immune sensor responsible for regulating the activation of caspase-1 and gasdermin D. A deeper understanding of pyroptosis and its interplay with other forms of regulated cell death is emerging, shedding light on a complex regulatory network controlling pore-forming proteins and cell fate. Cell death processes play a central role in diseases such as metabolic dysfunction-associated steatotic liver disease, metabolic dysfunction-associated steatohepatitis, autoinflammatory disorders, and cancer. Cell death often acts as a starting point in these diseases, making it an appealing target for drug development. Yet, the complete molecular mechanisms are not fully understood, and new discoveries reveal promising novel avenues for therapeutic interventions. In this review, we summarize recent evidence on pathways and proteins controlling pyroptosis and gasdermins. Furthermore, we will address the role of pyroptosis and the gasdermin family in metabolic dysfunction-associated steatotic liver disease and steatohepatitis. Additionally, we highlight new potential therapeutic targets for treating metabolic dysfunction-associated steatohepatitis and other inflammatory-associated diseases.

## 1 Introduction

Pyroptosis is a form of regulated cell death that depends on the formation of plasma membrane pores by members of the gasdermin (GSDM) protein family, often due to inflammatory caspase activation (Galluzzi et al., 2018). In the 1990s, pyroptosis was initially described as a caspase-1-dependent and bacteria-induced necrotic cell death; present in macrophages infected with *Salmonella typhimurium* (Zychlinsky et al., 1992; Monack et al., 1996). The term “pyroptosis” was coined by Cookson and Brennan in 2001 (from the Greek “*pyro*”, meaning fire or fever, and “*ptosis*”, denoting a falling) (Cookson and Brennan, 2001). Pyroptotic cell death is an important contributor to physiologic and pathologic processes dependent on the activation of the pore-forming gasdermin protein family. Once initiated, signaling cascades trigger the inflammasome-dependent and -independent formation of cell membrane pores, resulting in rupturing of the cell membrane, releasing its content such as inflammatory molecules and danger signals that promote inflammation and cellular infiltration. Besides its physiologic role in regulating cell death, pyroptosis has been associated with the pathogenesis of a variety of diseases, including metabolic dysfunction-associated steatohepatitis (MASH), formerly known as nonalcoholic steatohepatitis (NASH), inflammatory bowel disease (IBD), atherosclerosis, diabetes, and cancer (Rao et al., 2022). The regulation of cell death is a central part of physiologic cell development and homeostasis. The highly orchestrated homeostasis is severely impaired in dysregulated inflammation and chronic exposure to pathogenic or danger signals. Two different pathways regulate the mechanisms of pyroptosis, namely the canonical and the non-canonical pathway. The classical or canonical pathway includes the inflammasome activation and caspase-1-dependent cleavage of gasdermin D (GSDMD), whereas the non-canonical pathway initiates inflammasome-independent GSDMD cleavage via caspase-4/5/11. Cells undergoing pyroptosis can be observed exhibiting distinct morphological features such as chromatin condensation with an intact nucleus, mild cell swelling, membrane blebbing, the formation of pyroptotic bodies, and plasma membrane permeabilization, which is caused by the pore formation of cleaved gasdermins (Jorgensen and Miao, 2015; Yu et al., 2021). The pores lead to uncontrolled ion flux and the secretion of damage-associated molecular patterns (DAMPs) and proinflammatory cytokines intrinsic to pyroptotic cell death (IL-1β and IL-18). Recent advancements improved the understanding of the interactions between different upstream and downstream effectors of the inflammasome, which is at the center of the canonical pathway. Of high interest is the role of the pore-forming gasdermin family. Gasdermin activation however is not solely governed by canonical and non-canonical pyroptotic pathways, given the increasing evidence that multiple (cell death) pathways influence the activation or inhibition of the gasdermin family. Moreover, it is not completely understood when GSDMD activation only leads to the release of proinflammatory cytokines and DAMPs or irreversibly results in cell death. In this review, we will summarize new data on the interplay of pyroptosis and pathways regulating gasdermin activation, discuss their roles in MASH, and potentials as therapeutic targets.

## 2 Pyroptosis and gasdermins

### 2.1 Canonical pathway

**FIGURE 1:**
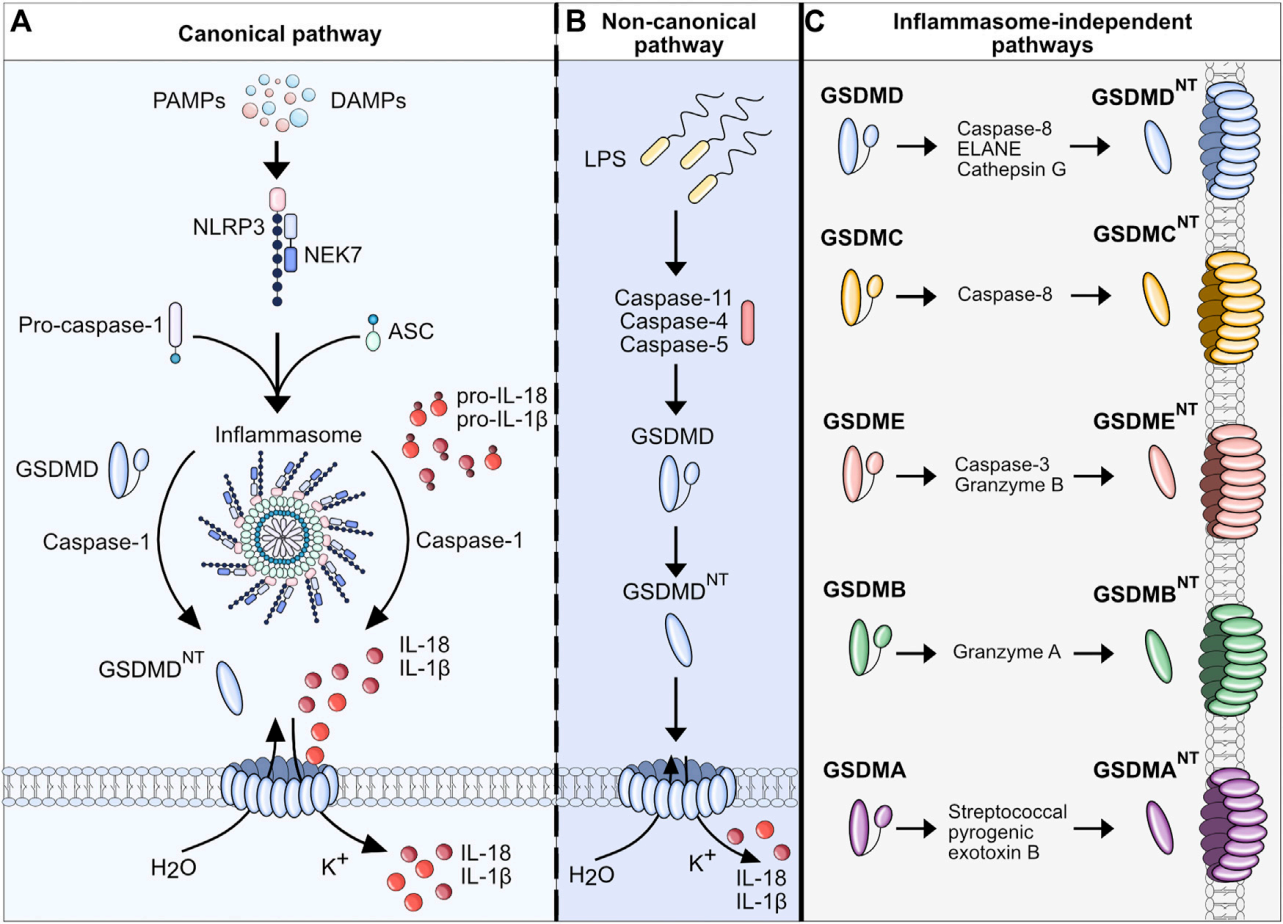
Pathways of pyroptosis and gasdermin activation. **(A)** Canonical pathway: Upon detection of DAMPs and PAMPs, inflammasome sensor NLRP3 recruits adaptor protein ASC to mediate CARD–CARD interactions with the effector cysteine protease caspase- 1. Active caspase-1 cleaves GSDMD into its NT fragment (GSDMD^NT^) and its auto-inhibitory C-terminal fragment. GSDMD^NT^ oligomerizes and forms membrane pores which induces pyroptotic cell death. Caspase-1 also cleaves pro-IL-1β and pro-IL-18 into their active forms, which are released through GSDMD pores. **(B)** Non-canonical pathway: Human caspases 4 and 5 and the murine orthologue caspase-11 are activated by direct and highly specific binding of intracellular LPS from Gram-negative bacteria. After binding LPS via the caspase-4/5/11 CARD domain, a complex, called the non-canonical inflammasome, is formed without NLRs or ASC. Pyroptosis is induced by the ability of the caspase-11 inflammasome to directly cleave GSDMD into pore-forming GSDMD^NT^. Proteolysis of pro-IL-1β and pro-IL-18 requires active caspase-1 which can be triggered by K+ efflux. **(C)** Inflammasome-independent pathways of pyroptosis: Different proteins and enzymes have been discovered that control gasdermins and pyroptosis. Control over GSDMD and GSDME via non-inflammatory caspases indicates overlapping cell death pathways and control mechanisms.

In the classical or canonical pathway, activation of the inflammasome is the key characteristic (Figure 1A). The inflammasome itself is a multi-protein complex composed of three main components: the sensor, the adaptor protein apoptosis-associated speck-like protein containing a CARD (ASC), and the effector caspase-1. Three different groups of inflammasome sensors are known that are categorized by their domain structures: The nucleotide-binding oligomerization domain (NOD)-like receptors (NLRs) that contain a leucine-rich repeat (LRR) domain (NLRC4, NLRP1, NLRP3, NLRP6), the absent in melanoma 2-like receptors (ALRs, AIM2-like receptors), which have a characteristic HIN 200 DNA-binding domain instead of a LRR, and the RIG-I-like receptor (RLR) family (Ireton and Gale, 2011; Schattgen and Fitzgerald, 2011; de Zoete et al., 2014). Among these, NLRP3 is the best described and studied inflammasome sensor. The canonical pathway ignition involves a two-step process consisting of the priming signal and the activation signal. The priming signal involves the activation of pattern recognition receptors (PRRs), mainly Toll-like receptors (TLRs), in response to pathogen-associated molecular patterns (PAMPs) or host-derived DAMPs (Bauernfeind et al., 2009; Franchi et al., 2009). Endotoxins, microbial DNA and RNA, flagellin, unsaturated fatty acids, host-derived ATP, high mobility group box 1 (HMGB1), uric acid, and heat shock proteins are among the DAMPs and PAMPs that induce the priming phase. Consequently, gene transcription of nuclear factor kappa B (NF-κB) target genes increases, which results in the upregulation of NLRP3, pro-IL-1β, and pro-IL-18 expression. During the priming phase, post-translational modifications render NLRP3 inactive but signal-competent (Swanson et al., 2019). It is not completely understood how NLRP3 senses the perturbation of homeostasis after priming through an extracellular signal. It is known that several upstream signaling pathways and their cellular signals, such as efflux of ions, mitochondrial dysfunction, and metabolic changes, translate the extracellular and intracellular detection of inflammatory mediators to the activation of NLRP3 inflammasome (Hornung et al., 2008; Zhang et al., 2010; Murakami et al., 2012; Munoz-Planillo et al., 2013; Triantafilou et al., 2013; Moon et al., 2016; Di et al., 2018; Green et al., 2018). However, not all of these pathways exclusively activate the inflammasome (Swanson et al., 2019). After the upregulation of inflammasome components during the priming phase and recognition of cellular stress in the activation phase, the inflammasome assembles by binding to the ASC adaptor. Thereafter, ASC forms helical filaments that merge into a large protein complex (Lu et al., 2014). ASC further recruits pro-caspase-1 via C-terminal caspase-recruitment domain (CARD)-interaction. Consequently, pro-caspase-1, a cysteine protease formerly known as ICE (interferon converting enzyme), is auto-activated by cleavage into its CARD domain and p20/p10 dimers. Active caspase-1 cleaves pro-IL-1β and pro-IL-18 into their active forms, IL-1β and IL-18, respectively. Besides processing proinflammatory cytokines, NLRP3 inflammasome activation can lead to lytic cell death. Current findings support a model in which caspase-1 mediates pore formation by cleaving GSDMD in its linker region and thereby liberating the cytotoxic 31-kDa N-terminal fragment (GSDMD^NT^) from its auto-inhibitory C-terminal fragment (GSDMD^CT^) (Kayagaki et al., 2015; Shi et al., 2015). GSDMD^NT^ binds to negatively charged lipids (e.g., phosphatidylinositide, phosphatidylserine, and cardiolipin) in the cell membrane inner leaflet, oligomerizes, and forms pores with inner and outer diameters of around 21–31 nm, containing 31–34 symmetric protomers (Ding et al., 2016; Liu et al., 2016; Sborgi et al., 2016; Xia et al., 2021). Evidence suggests that the GSDMD pore structure is dynamic, alternating between open and closed states and regulating pore size (Santa Cruz Garcia et al., 2022). GSDMD biochemically determines pyroptosis downstream of inflammasome activation. The finding was supported in murine models where mice lacking GSDMD had a significant reduction in IL-1β secretion and showed resistance to lipopolysaccharide (LPS)-induced septic shock (Kayagaki et al., 2015). The GSDMD pore serves two main purposes: it causes uncontrolled efflux of cytosolic electrolytes and organelles, leading to the death of the cell (pyroptosis), and secondly, releasing the aforementioned cytokines IL-1β and IL-18 via non-conventional secretion (Evavold et al., 2018). Upon release, IL-1β and IL-18 act as danger signals and perpetuate the inflammatory response of the innate and adaptive immune systems. Mature IL-1β mainly contributes to the activation and differentiation of neutrophils and monocytes, whereas interferon (IFN)-γ production and maturation of T_H1_ cells, NK cells, cytotoxic T cells, and T_H2_ cells are orchestrated by IL-18 (Mantovani et al., 2019).

### 2.2 Non-canonical pathway

The non-canonical pathway describes the activation of the human caspase-4 and -5 and the murine orthologue caspase-11 by cytosolic LPS independent of inflammasome activation (Figure 1B). The upregulation of caspase-4/5/11 is initiated by the binding of extracellular bacterial LPS to TLR4 (Kayagaki et al., 2015). Upstream mechanisms that regulate the binding of LPS to caspase-4/5/11 are not yet fully understood. It is known that the binding of LPS leads to the expression of small GTPases, guanylate-binding proteins (GBPs), which consequently lyse intracellular bacteria and liberate LPS to the cytosol (Meunier et al., 2014; Lee et al., 2018a). Upregulated caspase-4/5/11 detects intracellular LPS via its CARD domain and consequently forms the non-canonical inflammasome consisting of caspase-4/-5 or -11 and LPS (without ASC or NLRs) (Shi et al., 2014). Activated by auto-cleavage, caspase-4/5/11 cleaves and activates GSDMD, which leads to pore formation, secretion of proinflammatory cytokines and K^+^ efflux (Kayagaki et al., 2015). K^+^ efflux can further trigger the activation of caspase-1 which is required for the proteolytic processing of pro-IL-1β and pro-IL-18. The non-canonical pathway is negatively regulated by another subgroup of IFN-inducible GTPases, the immunity-related GTPase family M member 2 (Irgm2). Dysfunction of Irgm2 results in increased pyroptosis and a higher susceptibility to endotoxemia-induced lethality (Finethy et al., 2020). Caspase-11-dependent cell death in macrophages can also be initiated by the protein carboxypeptidase B1 (Cpb1), a complement-related protein, via the Cpb1–C3–C3aR signaling pathway in a mouse endotoxemia sepsis model (Napier et al., 2016).

### 2.3 New insights in the negative regulation of pyroptosis and cell death

Given the diverse signaling pathways in different cells controlling the gasdermins, pore formation can have cell death-dependent and -independent effects. For instance, induction of the NLRP3 inflammasome with various pathogens can elicit a GSDMD-dependent release of mature IL-1β from live murine macrophages in the absence of detectable cell lysis (Evavold et al., 2018). As the activation of inflammatory caspases leads to gasdermin processing, pyroptotic cell death is usually determined within seconds to minutes. However, recent evidence proposes that mammalian cells can initiate a cell-intrinsic membrane repair process via the mediator endosomal sorting complexes required for transport (ESCRT), which counters pyroptotic death execution (Ruhl et al., 2018). Interestingly, ESCRT-III-mediated repair of the plasma membrane also occurs in pore formation of MLKL-dependent necroptosis (Gong et al., 2017). However, the exact cellular mechanisms that fine-tune and modulate cell fate, after activation of cell death pathways, remain to be elucidated. It is intriguing to hypothesize that increasing intracellular levels of pore-forming proteins overcome counteracting mechanisms and induce cell death. New insights on the fine-tuning of the pore-formation in pyroptosis and other types of cell death were provided by Kayagaki et al. (2021). The cell-surface protein Ninjurin1 (NINJ1) is widely expressed, including in myeloid cells and macrophages. Murine *Ninj1* ^−/−^ and *Gsdmd* ^−/−^ bone-marrow derived macrophages (BMDMs) released less lactate dehydrogenase (LDH), a surrogate marker for cell death, upon stimulation with LPS, nigericin or infection with different bacterial pathogens. The pore formation of GSDMD as well as the release of proinflammatory cytokines IL-1β and IL-18 seem unaffected by NINJ1. The study delivers evidence that in macrophages NINJ1 prevents pyroptosis-induced cell membrane rupture which enables the release of IL-1β and IL-18 via GSDMD pores without undergoing lytic cell death. Further experiments showed that not only plasma membrane rupture is averted by NINJ1 but also the release of certain intracellular proteins such as HGMB1, a proinflammatory DAMP. However, Ninj1^−/−^ mice being more susceptible to infection with *C.rodentium* and wild-type (WT) BMDMs releasing proinflammatory cytokines would suggest an overall pro-inflammatory role for NINJ1. In a LPS-induced sepsis mouse model, *Ninj1* ^−/−^ mice showed similar survival to WT mice, whereas *Casp11* ^−/−^ mice showed a significantly improved survival, indicating that GSDMD activation without NINJ1 control leads to cell death. The study gave insights into the regulation of cell death-related cell membrane rupture beyond pyroptosis. In additional *in vitro* experiments *Ninj1*-deficient and WT BMDMs were stimulated with apoptosis- and necrosis-inducing agents and *Ninj1*-deficiency attenuated the release of intracellular proteins. Noteworthy, concomitant stimulation with TNF and the pan-caspase inhibitor zVAD, which induces MLKL-dependent necroptosis, resulted only in a partially reduced release of LDH whereas the release of intracellular proteins remained unchanged. This supports the conclusion that other NINJ1-unrelated mechanisms must take effect to negatively regulate membrane rupture in necroptosis. In a second study Kayagaki et al. (2023) treated WT mice with a NINJ1-antibody and used transgenic Ninj1^fl/fl^ *Rosa26*-CreER^T2^ mice in the setting of different hepatitis models to better understand the role of NINJ1 in tissue injury. Both, antibody treatment and the systemic deletion of *Ninj1* resulted in decreased levels of the liver enzymes alanine aminotransferase (ALT) and aspartate aminotransferase (AST) as well as LDH in serum. Interestingly, induction of hepatocyte apoptosis with TNF and the transcriptional inhibitor D-Galactosamine (D-Gal) lead to increased serum levels of HGMB1 and IL-18, which is rather known as a pyroptosis-related cytokine. In fact, TNF can induce proteolysis of IL-1 family cytokines to be biologically active via caspase-8 in macrophages and dendritic cells which could explain the increase of IL-18 in the applied hepatitis model (Bossaller et al., 2012). Distinct features and differences between cell deaths are described in Box 1. The effect of NINJ1 on tissue injury was further tested in a liver ischemia-reperfusion injury (IRI) model (Hu et al., 2023). Ninj1 expression was increased in both hepatocytes and Kupffer cells (KCs) of IRI-induced WT livers. Bone marrow chimeric mice that were deficient for Ninj1 in KCs displayed suppressed hepatic inflammation and neutrophil infiltration, both hallmarks of IRI-induced liver damage. The new insights improve our understanding of control mechanisms in cell death. However, the majority of the evidence comes from mechanistic studies in immune cells (mainly macrophages and neutrophils), and little is known regarding the role of pyroptosis and lytic cell death in other cell types and tissues such as epithelia, connective tissue, or endothelium that have been shown to express all components of the inflammasome and respond to both sterile and infectious stressors (Peeters et al., 2015).

BOX 1 **Different types of regulated cell death.**

**Apoptosis**

Apoptosis, a regulated cell death process, involves the activation of caspases. The extrinsic apoptotic pathway is typically initiated by perturbations in the extracellular microenvironment that are sensed by death receptors on the plasma membrane, propagated by caspase-8/RIPK1 and ultimately executed by the effector caspases-3 and -7. Intrinsic apoptosis is triggered by intracellular disturbances which lead to mitochondrial outer membrane permeabilization and the release of apoptogenic factors such as cytochrome *c*. Following the release of cytochrome *c*, a supramolecular complex known as apoptosome is formed and activates caspase-9 which in turn activates the executioner caspases caspase-3 and -7. New evidence suggests that apoptotic cells can undergo inflammatory post-apoptosis (secondary necrosis) and release DAMPs. Apoptosis is negatively regulated by cellular inhibitors of apoptosis (cIAPs) and inhibition of caspases can skew apoptosis towards necroptosis.

**Necroptosis**

Necroptosis is a type of regulated cell death that resembles features of apoptosis and necrosis. Necroptosis can be initiated by death receptors or other PRRs. The molecular signaling of necroptosis relies on the sequential activation of receptor-interacting protein kinases 1 and 3 (RIPK1 and 3) and mixed lineage kinase domain-like pseudokinase (MLKL). RIPK1 and 3 serve as cell stress sensors and recruit MLKL to a protein complex, called the necroptosome, which induces membrane lysis and release of DAMPs. NINJ1 only partially mediates plasma membrane rupture in necroptosis. ESCRT-III can antagonize necroptosis by shedding broken membranes into blebs.

**Pyroptosis**

Pyroptosis is a recently discovered form of regulated cell death that depends on the formation of plasma membrane pores by members of the gasdermin protein family, in particular GSDMD. Pyroptosis can be triggered by extra- and intracellular DAMPs and PAMPs alike. The canonical inflammasome activation via NLRP3 and caspase-1 triggers GSDMD cleavage, whereas the non-canonical cleavage of GSDMD is executed via murine caspase-11 and human homologue caspase-4/5. However, overlapping pathways with other forms of cell death exist, indicating the presence of a much more intricate and complex control system. Pore formation leads to cell membrane lysis and release of proinflammatory cytokines IL-1β and IL-18. Under certain circumstances, only proinflammatory cytokines and intracellular DAMPs are released.

## 3 The gasdermin family and activation—Control over cell fate

Gasdermins are expressed in various cell and tissue types and were initially identified in the murine gastrointestinal tract and epidermis, hence, their “gasdermin” nomenclature (Tamura et al., 2007). The six known members of the GSDM family (Box 2) are GSDMA, GSDMB, GSDMC, GSDMD, GSDME (also known as DNFA5), and PJVK (DFNB59). All gasdermins (except PJVK, which has a truncated C-terminal domain) share a similar two-domain architecture in which the C-terminal domain functions as an inhibitory domain to restrict the N-terminal pore-forming function. Both domains are connected through a linker region unique to each gasdermin protein. Beyond the first observations of GSDMD mediating caspase-1 and 11-dependent pyroptosis in immune cells, subsequent studies revealed many similar and unique features that characterize gasdermins, including their cellular source, activation pathways, and biological functions (He et al., 2015; Kayagaki et al., 2015; Shi et al., 2015).

BOX 2 **Gasdermin Family.**

**GSDMA**

GSDMA (also known as GSDM, GSDM1, or FKSG9) is mainly involved in the differentiation and homeostasis of epithelial cells of the oesophagus, stomach, bladder, and skin in humans (Saeki et al., 2000; Saeki et al., 2007). In mice, it was found to be expressed in epithelia and the skin, including the epidermis, hair follicles, and stomach (Tamura et al., 2007; Tanaka et al., 2013). Polymorphisms in GSDMA are associated with skin disorders and inflammatory bowel disease (Soderman et al., 2015; Terao et al., 2017). It was found to be suppressed in gastric cancer cells and was upregulated by TGFβ (Saeki et al., 2007).

**GSDMB**

GSDMB (also known as GSDML, PP4052, or PRO2521) is expressed in various tissues, but especially in the gastrointestinal epithelium/mucosa (Fagerberg et al., 2014). In addition, GSDMB is highly expressed in different cancer cell lines. In clinical tumor samples, GSDMB was prevalently expressed in colon, rectal, pancreatic, and cervical cancers. GSDMB is also highly expressed in differentiated airway epithelial cells, including the ciliated cells (Zhou et al., 2020). In a knockin mouse model, GSDMB induced pyroptotic cell death via the canonical pyroptosis pathway contributing to the pathogenesis of asthma (Panganiban et al., 2018). GSDMB is also described as having implications for IBD. Mechanistically, it was shown that apoptotic executioners caspase-3, -6, and -7 cleave and activate GSDMB (Chao et al., 2017). Further evidence showed that GSDMB activation was important for the restoration of epithelial restitution/repair in IBD (Rana et al., 2022). Besides the activation through the canonical and apoptotic pathways, lymphocyte-derived granzyme A cleaved GSDMB in human epithelial cells and led to a significant tumor clearance in co-treatment with an anti-PD-1-antibody in a murine tumor model (Zhou et al., 2020).

**GSDMC**

GSDMC was initially detected as a gene upregulated in metastatic murine melanoma, and its expression is evident in the oesophagus, skin, spleen, and vagina (Watabe et al., 2001; Fagerberg et al., 2014). It was further defined as a tumor suppressor gene in esophageal and gastric cancers (Saeki et al., 2009). In contrast, it was upregulated in colorectal cancer tissues and associated with increased tumor proliferation in a murine colorectal cancer model (Miguchi et al., 2016).

**GSDMD**

GSDMD (also known as GSDMDC1, DFNA5L, or FKSG10) is widely expressed in different human tissues (Rieckmann et al., 2017). Loss of GSDMD expression rescues mice with a D301N *Nlrp3* mutation causing the Neonatal-onset multisystem inflammatory disease (NOMID) as well as mice with a neutrophil-specific A350V *Nlrp3* mutation, which is associated with Muckle-Wells Syndrome (Xiao et al., 2018; Kaufmann et al., 2022a). The protection from systemic inflammation indicates that GSDMD activation is under the control of NLRP3. In addition to its other functions, GSDMD has a pleiotropic role in infection models. Following gastrointestinal murine norovirus infection, NLRP3 inflammasome-induced GSDMD cleavage and pyroptosis contributed to a more severe immunopathology, while GSDMD deficiency in mice prolonged their survival (Dubois et al., 2019). During murine bacterial infections, it was shown that the conserved type III secretion system (T3SS) rod proteins and LPS activate inflammasome-mediated pyroptosis in macrophages, releasing tissue factor that triggers coagulation and thrombosis, leading to death. However, in the absence of GSDMD, coagulation and lethality are prevented (Wu C. et al., 2019a). In an *E. coli*-peritonitis model, GSDMD deficiency improved host defense by delaying neutrophil cell death (Kambara et al., 2018). Gasdermin D proteins are recognized for their pyroptosis-independent functions in cancer cells. A recent study has identified lower expression of GSDMD in gastric cancer tissue, which correlated with increased cell proliferation in gastric cancer (Wang et al., 2018).

**GSDME**

GSDME (also known as ICERE-1 or DFNA5) was initially identified as a gene responsible for autosomal dominant non-syndromic hearing loss and was later found to possess sequence and structural similarities to the gasdermins (Van Laer et al., 1998; Op de Beeck et al., 2011). GSDME is variably expressed in different human cells and tissues, including the brain, endometrium, placenta, and intestine, among others. In mice and humans, GSDME is mainly processed by caspase-3, which induces post-apoptosis (or secondary necrosis) after cells undergo apoptosis (Rogers et al., 2017; Wang et al., 2017). In addition, caspase-independent cleavage and activation of GSDME are exerted by granzyme B, a protease that is released by cytotoxic T cells and NK cells. Granzyme B cleaves GSDME at the same site as caspase-3, releasing a N-terminal fragment that forms pores and induces cell death (Zhang et al., 2020). It is important to note that GSDME can also induce a non-lytic form of pyroptosis, where inflammatory cytokines are released without cell membrane disruption. Besides cell death, GSDME activation leads to the production and secretion of IL-1α and IL-1β from macrophages, IL-1β from neutrophils, HMGB1 from intraepithelial cells, and IL-18 from gastric cancer cell lines (Aizawa et al., 2020; Tan et al., 2020; Huang J. et al., 2021a; Chen et al., 2021; Zhou and Abbott, 2021). Expression levels of GSDME were increased in a human cohort with oesophageal squamous cell carcinoma and associated with significantly better overall survival, hence acting as a tumor suppressor (Wu M. et al., 2019a). Further evidence of its antitumor activity was delivered by Zhang et al. (2020) The authors showed that expression of GSDME in different cancer lines leads to antitumor killer cell toxicity and less tumor growth in a murine model.

**GSDMF**

Pejvakin (PJVK, also known as DFNB59 or GSDMF) was initially cloned from the human testis. PJVK expression is high in the testis, but it is also broadly expressed in other tissues, including the hair cells of the inner ear and other cells of the auditory system (Delmaghani et al., 2006; Collin et al., 2007). It is unknown how PJVK is activated. The N-terminal fragment shows no pore-forming activity *in vitro* (Ding et al., 2016).

The caspase cleavage site residues in the linker region vary among gasdermin family members, and not all gasdermins are cleaved by caspases (Figure 1C). Even though the role of GSDMD as the executioner of pyroptosis signaling is well described, GSDMD deficiency does not completely suppress pyroptosis, pointing out the various overlapping and redundant pathways (Kayagaki et al., 2015; Galluzzi et al., 2018). Upon closer examination of the available evidence regarding gasdermin regulation, it can be concluded that (1) the repertoire of pathways leading to gasdermin activation expands beyond the caspase family, (2) gasdermins function differently in different cell types based on the stimuli, (3) pyroptosis and caspase-pathways might be fully dependent on one another to regulate pore formation via different gasdermins.

Apoptotic cell death is executed by caspase-8 (extrinsic apoptosis) or caspase-9 (intrinsic apoptosis). Both initiator caspases, in turn, activate the executioner caspases-3 and -7 (46). Recent evidence showed that gasdermin activation is not always caspase-1-dependent but is also regulated by known apoptosis-regulating caspases. Caspase-3, for instance, was identified to cleave GSDME in the linker region to liberate GSDME^NT^, which activates pyroptosis in a similar mechanism to that of GSDMD^NT^. In addition, caspase-3 was also shown to cleave GSDMD; however, cleavage occurs within the N-terminal region rather than in the linker region, leading to the inhibition of GSDMD by generating an inactive NT fragment (Rogers et al., 2017; Wang et al., 2017; Shojaie et al., 2020). In virus-infected macrophages, caspase-3-mediated cleavage of GSDME was observed to induce pyroptosis and cell rupture after cells already have entered apoptosis, which has been termed “secondary necrosis” (or post-apoptosis) (Rogers et al., 2017). In contrast, Wang et al. (2017) postulated that the caspase-3-GSDME axis can directly induce pyroptotic cell death in chemotherapy-treated cells lacking apoptotic features. Inhibition of caspase-1 as the main regulator of GSDMD activation seems logical to prevent pyroptotic cell death. However, it is likely that even when caspase-1 is inhibited, cells remain doomed by activation of compensatory signaling pathways. In the absence of caspase-1, cells can still undergo lytic cell death. In murine caspase-1-deficient BMDMs treated with flagellin, a bacterial PAMP and activator of the NLRC4 inflammasome, cell death was induced via NLRC4/caspase-8, resulting in apoptosis, plasma cell membrane damage, and subsequent secondary necrosis (Lee et al., 2018b). This cell death (of an unknown program) was independent of the GSDME-caspase-3 axis. The gasdermin activation via caspase-8 seems to be cell-specific: In response to the *Yersinia bacteria* infection, caspase-8 directly cleaves GSDMD, whereas GSDME is dispensable for macrophage cell lysis downstream of the ripoptosome (Chen et al., 2019). The authors proposed that—under the control of the ripoptosome (RIPK1 and RIPK3)-caspase-8-axis, which can control necroptosis and apoptosis—NLRP3 inflammasome activation was induced by potassium efflux through the pannexin-1 channel rather than GSDMD (Chen et al., 2019), which is in contrast to a previous study that showed that ion efflux and NLRP3 activation were caspase-8-dependent (Orning et al., 2018). However, pore formation via pannexin-1 would explain why GSDMD deficiency does not completely abolish IL-1β secretion. Apoptosis is negatively regulated by X-linked inhibitor of apoptosis (XIAP). Loss of XIAP increased cell death and inflammatory responses in both the colonic mucosa and peripheral blood mononuclear cells (PBMCs) of XIAP-deficient IBD patients reflecting heightened caspase-8, GSDMD, and IL-1β activation (Hughes et al., 2023). It appears that the current studies have only scratched the surface of our comprehension of the intricate mechanisms that regulate programmed cell death, which is essential for a controlled immune response and the prevention of adverse effects caused by an uncontrolled inflammatory response. Gasdermins, which are expressed in various cell types, have diverse functions depending on the cell type and pathological condition. Briefly, GSDMD activation in macrophages induces the release of proinflammatory cytokines and the release of GSDMD^NT^ fragments that can bind to bacterial cardiolipin leading to the rupture of bacterial membranes and efficient host defense. In neutrophils, the canonical, caspase-1-mediated activation of GSDMD induces pyroptosis but is dispensable for NETosis, a neutrophil-specific cell death characterized by the production of neutrophil extracellular traps (NETs) (Chauhan et al., 2022). Conversely, caspase-1-independent cleavage of GSDMD by either neutrophil-specific serine protease, neutrophil elastase (ELANE), or non-canonical caspases of the inflammasome was shown to be involved in NETosis by possibly forming membrane pores and allowing extrusion of DNA fragments that can form NETs (Chen et al., 2018; Kambara et al., 2018; Sollberger et al., 2018; Burgener et al., 2019). Of note, in an *E.coli*-induced peritonitis model GSDMD deficiency led to improved host defense by delaying neutrophil death and increased levels of proinflammatory cytokines in the peritoneal cavity, showing an anti-inflammatory effect of ELANE-dependent GSDMD activation in neutrophils (Kambara et al., 2018). Thus, GSDMD can play a pleiotropic and context-dependent role, exerting both pro- and anti-inflammatory effects. Further insights into the gasdermin pathway regulation in neutrophils were gained by the study of Burgener et al. (2019) The authors showed that CathepsinG activates both executors of apoptosis and pyroptosis, namely caspase-7 and GSDMD.

## 4 Pyroptosis and gasdermins in steatohepatitis

The incidence of metabolic dysfunction-associated steatotic liver disease (MASLD), formerly known as nonalcoholic fatty liver disease, is increasing worldwide (Younossi et al., 2018). The disease is complicated by its potential progression as a result of liver inflammation and fibrosis, termed metabolic dysfunction-associated steatohepatitis (MASH, formerly NASH), which increases the risk for the development of cirrhosis, liver failure, or the development of primary liver cancer. Approximately 10% of MASLD patients will progress to MASH, and 20%–25% of these patients will develop fibrosis that will progress to cirrhosis (Younossi et al., 2018). MASH is diagnosed via liver biopsy and is pathologically characterized by lobular inflammation, accumulation of intracellular lipids (steatosis), and hepatocyte ballooning, with or without fibrosis. However, the molecular and inflammatory mechanisms causing the progression to MASH are not yet fully understood. Hepatocyte cell death as a result of lipotoxicity is one of the key drivers that causes the progression of MASLD to MASH. The persistent release of DAMPs by stressed and injured hepatocytes is recognized by immune cells and can lead to chronic inflammation. However, cell death in the setting of MASLD/MASH is not restricted to hepatocytes but also occurs in various subsets of immune cells and other non parenchymal liver cells which can influence disease progression (Luedde et al., 2014). For a detailed description of different types of cell death in MASLD/MASH we refer the reader to extensive reviews on the topic as we will focus on pyroptosis and gasdermins in MASH (Schuster et al., 2018; Schwabe et al., 2020). Here, we will describe the role of NLRP3 and GSDMD in liver inflammation and MASH and discuss cell type-specific effects of pyroptosis in murine and human MASH.

In the liver, the first demonstration of pyroptotic cell death as a novel form of cell death contributing to tissue injury came from a study using *Nlrp3* knockin mice expressing the D301N *Nlrp3* mutation (ortholog of D303N in human *NLRP3*), resulting in a hyperactive NLRP3 (Wree et al. 2014a). NLRP3 activation resulted in shortened survival, poor growth, and severe liver inflammation in these mice. In the setting of diet-induced liver inflammation and fibrosis, levels of inflammasome components, including NLRP3, pro-IL-18, pro-IL-1β, ASC, and caspase-1 were found to be elevated in both MASH patients and experimental MASH animal models (Wree et al., 2014b; Gaul et al., 2021). These results indicated that NLRP3 is also a driver platform of inflammation in MASLD and facilitates progression to MASH or worsens MASH. However, studies using models of isolated steatosis initially suggested that at these early stages of the disease, global inhibition of inflammasomes may be detrimental to the liver. Indeed, Henao-Mejia et al. (2012) found that in models of isolated hepatic steatosis, including a short-term (4 weeks) methionine and choline-deficient diet (MCD) diet model, various global inflammasome-related knockouts (*Asc^−/−^*, *Caspase-1^−/−^*, *Il-18^−/−^*, *Nlrp6^−/−^*, *Nlrp3^−/−^*, *Nlrc4^−/−^*) had increased levels of ALT and AST as well as increased MASLD activity scores, with similar results in the leptin-receptor deficient animals (db/db) MASLD mouse model which was additionally co-housed with *Asc^−/−^* mice resulting in comparable levels of liver injury. Using murine models associated with more advanced diseases resembling human MASH and fibrosis, Wree et al. (2014b) demonstrated that the global *Nlrp3* knockout mice were protected from MASH-associated hepatomegaly, liver injury, and infiltration of activated macrophages. Further, hepatic fibrosis was attenuated but steatosis development seemed to be unaffected. In a second mouse model, a short-term choline-deficient L-amino-defined (CDAA) diet was fed for 4 weeks to induce steatosis in WT mice and tamoxifen-inducible *Nlrp3* knockin mice with the alanine 350 to valine (A350V) mutation. WT animals showed isolated hepatic steatosis while inducible *Nlrp*3 knockin mice showed severe liver inflammation, with increased infiltration of activated macrophages and early signs of liver fibrosis (Wree et al., 2014b).

Since GSDMD is identified as the executor of pyroptosis by forming pores in the cell membrane, it has been intriguing to consider its crucial, proinflammatory role in the development of MASH. As suspected, protein levels of GSDMD and its pyroptosis-inducing fragment GSDMD^NT^ were increased in liver tissues of human MASLD/MASH compared to healthy controls (Xu et al., 2018). The GSDMD^NT^ levels correlated with both the MASLD activity score and liver fibrosis. In the same study, GSDMD-deficient mice were either fed MCD to induce MASH or high-fat diet (HFD) to induce MASLD. In both models, GSDMD deficiency safeguarded the mice from steatohepatitis and fibrosis. Further, it was demonstrated that GSDMD triggered the expression of proinflammatory cytokines (such as IL-1β, TNF-α, and MCP-1), resulting in the activation of the NF-κB signaling pathway and the subsequent recruitment of macrophages in MASH, implying that the pyroptosis-GSDMD axis plays a crucial role in the development of MASH. Hepatocytes isolated from C57BL/6J mice fed a high-fat, high-cholesterol diet (HFHCD) for 12 weeks showed increased levels of GSDMD^NT^, suggesting that pyroptosis in hepatocytes reflects diet-induced liver injury (Koh et al., 2021). In addition, the knockdown of the NLRC4 inflammasome prevented MASH and significantly decreased GSDMD^NT^ levels. Interestingly, isolated hepatocytes from HFHCD-fed *Nlrp3* knockout mice in the same study had increased levels of GSDMD^NT^ fragments and increased cell death, whereas *in vivo* experiments indicated that caspase-1 and *Nlrp3* knockout mice were protected from HFHCD-induced hepatic inflammation and fibrosis, which is in line with previous results (Dixon et al., 2012; Mridha et al., 2017). The authors concluded that NLRP3 does not directly mediate hepatocyte-specific GSDMD-dependent pyroptosis, and the beneficial effects of NLRP3 inhibition are rather the results of its effects dampening inflammation triggered by infiltrating pro-inflammatory macrophages. In a diet model where mice were either fed a high-fat diet or additionally received the carcinogen 9,10-dimethyl-1,2-benzanthracene (DMBA) for hepatocellular carcinoma development, cleaved-caspase-1, caspase-11, and cleaved-GSDMD were upregulated in freshly isolated hepatic stellate cells (HSCs) (Yamagishi et al., 2022). A transient knockdown of GSDMD in cultured murine HSCs reduced the release of IL-1β, whereas the stimulation with lipoteichoic acid (TLR2 ligand) facilitated the expression and maturation of caspase-11, leading to the creation of N-terminal fragments and pore formation as well as increased expression of IL-1β and IL-33. The diet-induced MASH mouse models are summarized in Table 1. The pleiotropic effects of the inflammasome-gasdermin signaling axis in different stages of the MASLD-MASH sequence need further elucidation in future studies. All studies discussed above have in common that the transgenic mice were full-body knock outs and not cell type- or liver-specific. Given the cell-dependent effects of pyroptosis, it is of utmost importance to look at the cell type-specific role of NLRP3 in liver inflammation and MASH to understand such mechanisms better.

**TABLE 1:** Pyroptosis in diet-induced MASH/MASLD mouse models.

Transgenic mouse	Cell type	Diet model	Pathology	Ref.
Systemic *Nlrp3* deletion				
*Nlrp3* KO	Global	MASH: CDAA (16 weeks) *vs*. CSAA	Hepatomegaly ↓ Steatosis ↔	Wree et al. (2014b)
			Hepatic inflammation ↓ Liver fibrosis ↓	
		MASH: HFHCD (12 weeks)		Koh et al. (2021)
*Nlrc4* KO	Global	MASH: HFHCD (12 weeks)	Steatosis ↔ Liver fibrosis ↓ Hepatic inflammation ↓	Koh et al. (2021)
*Caspase-1* KO	Global	MASH: HFHCD (12 weeks), HFD (12 weeks), MCD (6 weeks)	Steatosis ↓ Liver fibrosis ↓ Hepatic inflammation ↓	Dixon et al. (2012), Dixon et al. (2013), Koh et al. (2021)
*Gsdmd* KO	Global	MASLD/early MASH: MCD (4 weeks)	Steatosis ↓ Hepatic inflammation ↓	Xu et al. (2018)
		Fibrosing MASH: MCD (8 weeks)	Liver fibrosis ↓ Hepatic macrophage infiltration ↓	
		MASLD/MASH: HFD (4, 11, or 36 weeks)	Steatosis ↓	
Cell-specific *Nlrp3* deletion				
*Nlrp3* ^fl/fl^Lyz-Cre	Myeloid cells	MASH: CDAA-HFD (10 weeks)	Hepatic inflammation ↓ Liver fibrosis ↓	Kaufmann et al. (2022b)
		MASH: Western Diet (20 weeks)		

KO, knockout; MASH, metabolic dysfunction-associated steatohepatitis; CDAA, choline-deficient L-amino acid defined; HFHCD, high-fat, high-cholesterol diet; HFD, high-fat diet; MCD, choline-deficient diet; MASLD, metabolic dysfunction-associated steatotic liver disease.

**FIGURE 2:**
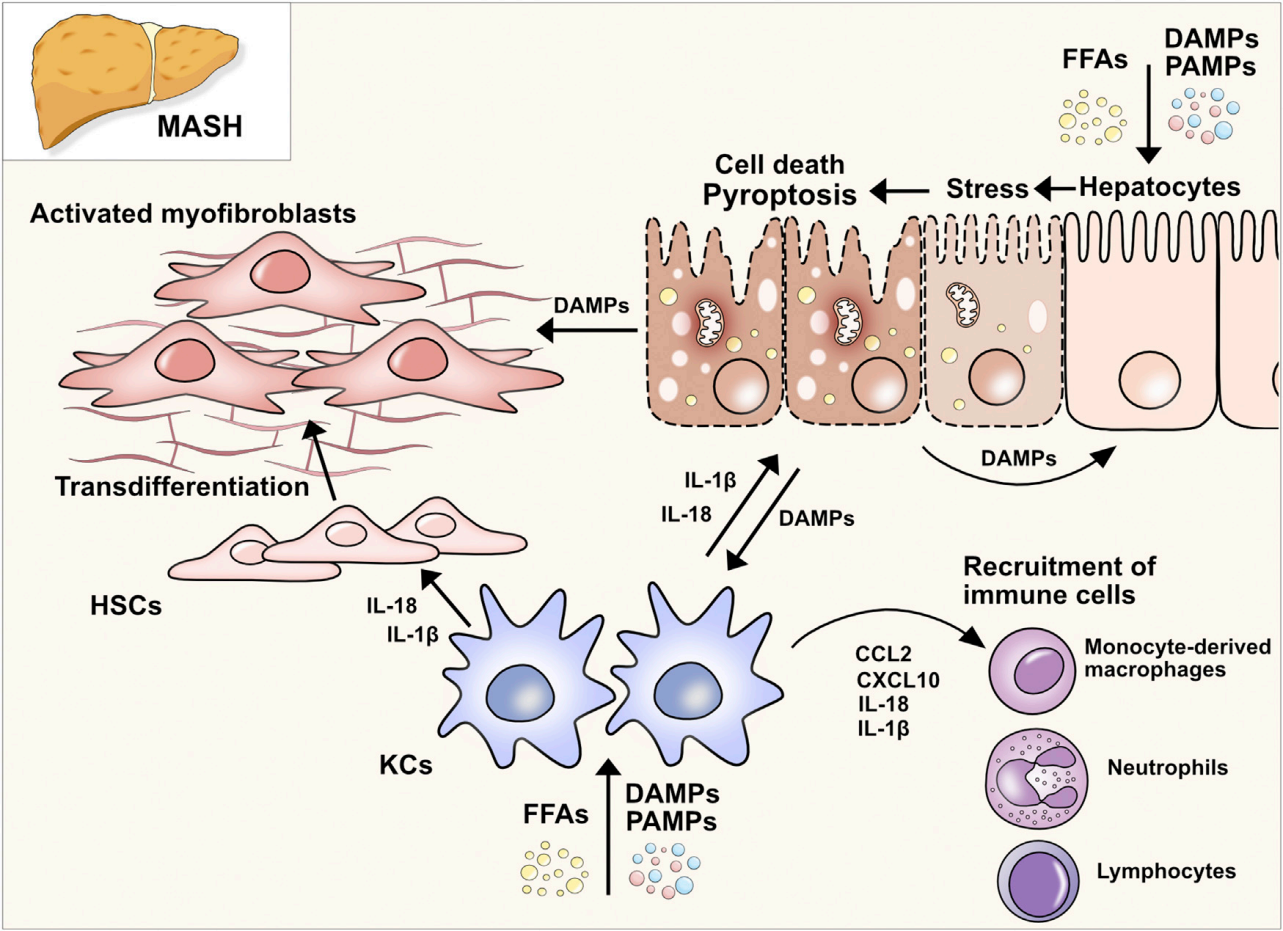
Pyroptosis in MASH. Upon MASLD-associated metabolic and inflammatory changes, hepatocytes are constantly exposed to an increased concentration of free fatty acids (FFAs), DAMPs, and PAMPs, eventually triggering cell death, in particular pyroptosis. The release of intracellular DAMPs, such as mitochondria, host RNA and DNA, NLRP3 components, and mature IL-1β and IL-18, further induces pyroptosis in neighboring hepatocytes and nonparenchymal cells. IL-1β and IL-18 can selectively stimulate the transdifferentiation of quiescent HSCs to collagen-producing myofibroblasts. The uptake of extracellular NLRP3 components can also activate HSCs and facilitate fibrotic changes in the liver (Gaul et al., 2021). Activated HSCs transdifferentiate to myofibroblasts which promotes fibrogenesis and further maturation of proinflammatory interleukins, perpetuating the detrimental effects of cell death and inflammation. In resident macrophages (Kupffer cells, KCs) the imbalance of lipid homeostasis contributes to a proinflammatory polarization and enhances inflammation in MASLD. In addition, the extracellular DAMPs of stressed and dying hepatocytes perpetuate hepatic inflammation by stimulating KCs to secrete multiple proinflammatory and immune cell attracting cytokines (e.g. CCL2, CXCL10). Ultimately, this interplay leads to the infiltration of various subsets of immune cells, which can either exacerbate or resolve the condition of MASH.

Apart from different disease stages, recent studies have allowed for a better understanding of pyroptosis and inflammasome activation in MASLD/MASH by focusing on the cell-specific effects of pyroptosis. Liver inflammation and fibrogenesis in MASH are complex and dynamic processes, necessitating intricate interplays amongst diverse cell populations, cytokines, and signaling pathways. The liver consists of hepatocytes as well as nonparenchymal cells including HSCs, liver sinusoidal endothelial cells (LSECs), Kupffer cells, and other immune cells. Recent evidence has shed light on the distinct roles played by these various cell types in MASH pathogenesis, unveiling specific subsets within immune cells that can exert varying effects on the disease. The detailed mechanisms of cellular interactions governing inflammation and fibrosis in steatotic liver disease have been extensively reviewed in other sources (Peiseler et al., 2022). Here we will focus on the cell type-specific-effect of pyroptosis in liver inflammation and MASH (Figure 2).

To investigate the cell-specific impact of NLRP3, Gaul et al. used a hepatocyte-specific *Nlrp3* ^L351P/+^Alb-Cre knockin mouse. It was shown that hepatocytes may undergo NLRP3-mediated pyroptosis and thereby amplify inflammasome-driven hepatic fibrogenesis (Gaul et al., 2021). The study also added to the knowledge on pyroptosis-related intercellular communication by uncovering that HSCs engulf extracellular NLRP3 inflammasome particles, increasing IL-1β secretion and α-smooth muscle actin expression in these cells. HSCs are known to be responsible for extracellular collagen deposition and play a central role in liver fibrogenesis. The role of pyroptosis in HSCs was further elucidated by an overexpression model of NLRP3. In the study by Inzaugarat et al. (2019) HSC-specific *Nlrp3* ^L351P/+^ knockin mice were generated and histological examination revealed increased collagen deposition in the liver as well as increased markers of fibrosis compared to WT littermates, indicating that pyroptosis can stimulate the transdifferentiation of quiescent HSCs to collagen-producing myofibroblasts. Markers for activation of macrophages and neutrophils were similar between *Nlrp3* ^L351P/+^Lrat-Cre knockin mice and WT. Neutrophils are also an abundant cell type in MASH livers and play a dual role in the pathobiology of MASH. They can trigger liver injury and fibrosis associated with diet-induced MASH in mice, presumably by releasing toxic molecules including proteases, oxidants, cytokines and NETs but depletion in the resolution phase of MASH leads to defective repair and remodeling processes (Kim et al., 2021). Their role as resolving effector cells in liver fibrosis that induce pro-inflammatory macrophages into a restorative phenotype and thereby reversing inflammation once the injury trigger ceases is potentially exerted via miR-223, a critical negative regulator of NLRP3 (Calvente et al., 2019). However, the role of NLRP3 activation in neutrophils during liver inflammation and fibrosis is not completely understood and available data is scarce. To shed light on these mechanisms, our group has developed mouse models with neutrophil-specific mutant NLRP3 (Kaufmann et al., 2022a). Mutant mice developed severe liver inflammation and lethal autoinflammation that was comparable to mice with a systemic expression of mutant NLRP3. NLRP3 activation in neutrophils leads to a pro-inflammatory cytokine and chemokine profile in the liver, infiltration by neutrophils and macrophages, and an increase in cell death. Furthermore, mutant mice develop liver fibrosis associated with increased expression of pro-fibrogenic genes. Details of the different pyroptosis-inducing models can be found in Table 2. Even though these studies gave broad insight into the cell-specific role of pyroptosis in liver fibrosis and inflammation, comprehensive data for the cell-specific impact of NLRP3 on MASLD/MASH is still scarce. To that end, Kaufmann et al. (2022b) compared mice that either had a hepatocyte-, HSC- or myeloid cell-specific deletion of *Nlrp3* and were fed a MASH-inducing diet (CDAA-HFD) or a Western diet. *Nlrp3* deletion in myeloid-derived cells led to reduced liver fibrosis and reduced markers for HSC activation (*Col1a, Col3a*). In contrast, the deletion of *Nlrp3* in HSCs or hepatocytes was not protective. In co-culture experiments, NLRP3 was activated in WT and *NLRP3* KO THP-1 cells (human monocytes), by stimulation with LPS and nigericin and then co-cultured with human LX-2 cells (HSCs). The HSCs were activated by the inflammasome-stimulated monocytes, and this effect was significantly reduced where NLRP3 was downregulated in monocytes. The findings highlight the pyroptosis-related crosstalk between monocytes and HSC contributing to the transdifferentiation of HSCs into active myofibroblasts.

**TABLE 2:** Pyroptosis in NLRP3-overexpressing mouse models.

Transgenic mouse/Nlrp3 mutant	Cell type	Pathology	Ref.
*Nlrp3* ^L351P/+^Alb-Cre	Hepatocytes	Hepatic inflammation ↑ Cell death ↑ Liver fibrosis ↑	Gaul et al. (2021)
*Nlrp3* ^D301N/+^Lyz-Cre	Myeloid cells	Hepatic inflammation ↑ Cell death ↑ Liver fibrosis ↑	Brydges et al. (2009), Wree et al. (2014a)
*Nlrp3* ^A350V/+^Lyz-Cre			
*Nlrp3* ^L351P/+^Lrat-Cre	Hepatic stellate cells	Hepatic inflammation ↔ Cell death ↔ Liver fibrosis ↑	Inzaugarat et al. (2019)
*Nlrp3* ^D301N/+^MRP8-Cre *Nlrp3* ^A350V/+^MRP8-Cre	Neutrophils	Hepatic inflammation ↑ Cell death ↑ Liver fibrosis ↑ Immune cell infiltration ↑	Kaufmann et al. (2022a), Chauhan et al. (2022)
*Nlrp3* ^A350V/+^Fcgr1-Cre	Macrophages	Hepatic inflammation ↑ Cell death ↑	Frising et al. (2022)

## 5 Inflammasomes and gasdermins as therapeutic targets in MASH

Great efforts have been made to develop compounds and drugs to inhibit key drivers of the pyroptosis pathways. The inflammasomes (mainly NLRP3) and the gasdermins are at the focus of drug development for various inflammatory diseases, including MASH. Considering the crucial role of GSDMD in inflammasome-mediated cell death and cytokine secretion, the blocking of gasdermin pores is currently being explored as a new therapeutic target for anti-inflammatory interventions. Regarding the GSDM family, Liu et al. (2019) analyzed mutations interfering with the lipid binding and oligomerization of gasdermins and thereby impeding cell lysis. These regions are responsible for lipid binding, oligomerization, and pore formation, making them appealing targets for developing therapeutic molecules through structure-based design. However, as for MASH, the development of a therapy has been challenging and no drugs have been approved for the treatment of the disease. Drug approval for MASH treatment requires a substantial histological improvement, a resolution of MASH and/or an improvement in fibrosis stage proven by liver biopsy. Seeing as fibrosis is an important predictor of morbidity and mortality outcomes and since pyroptosis contributes to hepatic inflammation and fibrosis, targeting pyroptosis (amongst other pro-inflammatory and profibrotic pathways) can be a therapeutic option. It should be mentioned that a variety of drugs directed against metabolic, proinflammatory and pro-fibrotic pathways associated with MASH are under investigation and are described in detail elsewhere (Tacke et al., 2023). In the following sections, we will highlight the current status and recent advances in the development of antipyroptotic therapeutics.

### 5.1 NLRP3

The NLRP3 inflammasome acts as a central signaling platform of the innate immune system and regulates the immune response in many infectious and non-infectious diseases. Hence, multiple NLRP3-specific inhibitors have been developed to inhibit the orchestrator of inflammation. Current NLRP3 inhibitors tested in clinical trials for various diseases are summarized in Table 3. NLRP3-inhibitors have been developed and tested in MASH models since cell death and pyroptosis play crucial roles in the progression from benign MASLD to inflammatory MASH. In different preclinical, diet-induced MASH mouse models, several groups have shown that the application of NLRP3 inhibitors (MCC950 or IFM-514) suppressed the severity of hepatic inflammation and fibrosis (Mridha et al., 2017; Torres et al., 2021). MCC950 (also known as CP-456 or CRID3) is a potent and selective small molecule inhibitor of NLRP3 that blocks the ability of NLRP3 to hydrolyze ATP for NLRP3 inflammasome function (Coll et al., 2015; Coll et al., 2019). Torres et al. (2021) tested the NLRP3-inhibiting compound IFM-514 in ApoE^−/−^ mice on an MCD or Western diet and demonstrated therapeutic effects against steatohepatitis by decreasing hepatic inflammation and fibrosis. The lipid accumulation in the liver was only minimally affected, suggesting that NLRP3 inhibition prevents inflammation in MASH, but does not target metabolic changes. However, even though MCC950 and IFM-514 were proven effective in preclinical models, no clinical trials for MASH have begun. Apart from NLRP3 inhibitors that specifically target the NLRP3 inflammasome, some pleiotropic therapeutic agents exist that also attenuate by inhibiting NLRP3-mediated pyroptosis. One such drug is Liraglutide, a glucagon-like peptide-1 (GLP-1) receptor agonist, which is used as a therapeutic in type 2 diabetes treatment also has a broad anti-inflammatory effect. Liraglutide increases pancreatic insulin secretion, regulates appetite, and can induce weight loss. In a murine MASH model, Liraglutide reduced lipid accumulation, inhibited NLRP3 inflammasome and pyroptotic activation, and attenuated mitochondrial dysfunction and reactive oxygen species (ROS) generation. Furthermore, it augmented mitophagy in hepatocytes (Yu et al., 2019). However, there is limited data on the effects of GLP-1 agonists/receptors on immune cells and cell death, and the effects on liver fibrosis are thought to be mainly indirect by improving hepatocyte metabolism. In a 2016 phase II trial published by Armstrong et al. (2016), it was demonstrated that in a small patient cohort of 52 individuals, Liraglutide was safe, well-tolerated, and resulted in histological resolution of MASH. A long-acting version of Liraglutide, Semaglutide, which also shows NLRP3 inhibition, was tested in a double-blind phase 2 trial including patients with biopsy-confirmed MASH and liver fibrosis of stage F1, F2, or F3 (Wang et al., 2021a; Newsome et al., 2021). Treatment with semaglutide resulted in a significantly higher percentage of patients with MASH resolution than treatment with placebo. Semaglutide is currently under investigation in a phase 3 randomized controlled trial (RCT) (ESSENCE trial, NCT04822181); enrolling more than 1,200 patients to examine its effect on the resolution of MASH in a broader context. Another promising drug that is tested for the treatment of MASH is obeticholic acid (OCA), a modified, synthetic bile acid, which acts as a farnesoid X-activated receptor (FXR) agonist and has additional anti-inflammatory effects. It selectively binds and activates the FXRs of enterocytes and hepatocytes, thereby reducing toxic levels of bile acids (Krupa et al., 2023). In several, preclinical MASH mouse models, the treatment with OCA improved steatosis and inflammation by inhibiting NLRP3-mediated pyroptosis (Yang et al., 2017; Huang et al., 2021b). Accordingly, the level of IL-1β and IL-18 in the liver, the hepatic expression of ASC, pro-caspase-1, and cleaved caspase-1 in liver macrophages were reduced. Interestingly, OCA inhibited NLRP3 inflammasome activation in BMDMs that were deficient for FXR, indicating that OCA acts independently of FXR to inhibit NLRP3. OCA has been proven effective in patients with non-cirrhotic MASH who were included in a phase 3 RCT (FLINT, NCT01265498) (Neuschwander-Tetri et al., 2015). The primary outcome was an improvement in the MASLD activity score by at least 2 points without worsening of fibrosis which was met in 35 (43%) out of 82 in the OCA group vs. 17 (21%) out of 82 in the placebo group. Long-term effects of OCA are currently being investigated for non-cirrhotic MASH in a phase 3, double-blind RCT (REGENERATE, NCT02548351) (Ratziu et al., 2019). The interim analyses after 18 months of treatment showed that the primary endpoint, fibrosis improvement (≥1 stage) with no worsening of MASH, was achieved by 23% of patients with fibrosis stage 2 or 3 who received OCA 25 mg *versus* 12% of patients who received placebo (*p* = 0.0002) (Younossi et al., 2019). Further analyses showed that non-invasive biomarkers for MASH were sustainably reduced and health-related quality of life was improved in patients with OCA treatment vs. placebo (Rinella et al., 2022; Younossi et al., 2022). Metabolism, inflammation and fibrogenesis in MASH are intricately entwined and inhibition of the NLRP3 inflammasome may be effective but would require a therapeutic approach that either targets pyroptosis and other signaling pathways simultaneously or involves a combination of different drugs at once to treat metabolic and inflammatory damage. One such approach was tested a phase II RCT in patients with MASH (NCT03987074). The phase II study examined the safety and efficacy of semaglutide alone and in combination with the FXR agonist cilofexor and/or the acetyl-coenzyme A carboxylase inhibitor firsocostat (Alkhouri et al., 2022). FXR agonist cilofexor is known to inhibit lipogenesis, gluconeogenesis, and bile acid synthesis whereas firsocostat reduces hepatic *de novo* lipogenesis. The combined treatment resulted in additional improvements in liver steatosis and attenuated liver injury indicated by lower levels of ALT, AST, and inflammatory markers such as CRP or CK-18 when it was compared to semaglutide alone.

**TABLE 3:** Overview of clinical NLRP3-specific inhibitor trials.

NLRP3 inhibitor	Clinical trial	Phase	Targeted Indication	Status/Results	Ref.
DFV890	NCT04382053	Phase II	COVID-19 pneumonia	No effect on disease severity	Madurka et al. (2023)
DFV890	NCT04868968	Phase II	FCAS	Recruitment completed	
DFV890	NCT04886258	Phase II	Knee osteoarthritis	Recruitment ongoing	
Inzomelid (IZD174)	NCT04086602	Phase I	Healthy subjects, CAPS	Recruitment completed	
Somalix (IZD334)	NCT04015076	Phase I & IB	Healthy subjects, CAPS	Recruitment completed	
NT-0249	Not specified	Phase I	Healthy subjects	No information available	
NT-0796	ACTRN12621001082897	Phase I	Healthy subjects	No information available	
ZYIL1	NCT04972188	Phase I	Healthy subjects	Recruitment completed	
Dapansutrile (OLT1177)	NCT03534297	Phase IB	HFrEF	Improved LVEF	Wohlford et al. (2020)
Dapansutrile	EudraCT 2016-000943–14	Phase IIA	Acute Gout flares	Reduced joint pain	Kluck et al. (2020)
HT-6184 (NEK7 inhibitor)	NCT05447546	Phase 1	Healthy subjects	Recruitment ongoing	

FCAS, familial cold autoinflammatory syndrome; CAPS, cryopyrin-associated autoinflammatory syndromes; HFrEF, heart failure with reduced ejection fraction; LVEF, left ventricular ejection fraction.

### 5.2 Caspases

Inflammatory and non-inflammatory caspases link the cellular detection of DAMPs and PAMPs to executioner caspases and pore-forming proteins that induce cell death. Multiple caspase inhibitors are available and have also been shown to reduce cell death and fibrosis in MASH mouse models (Dhani et al., 2021). However, only a few have reached clinical trials for MASH treatment. One of these is Emricasan, an oral pan‐caspase inhibitor that decreases apoptosis, inflammation, fibrosis, cirrhosis, and cell death in animal models of acute hepatitis and chronic models of MASH (Barreyro et al., 2015; Gracia-Sancho et al., 2019). While initial results of two phase 1 trials showed short-term improvements of ALT/AST, CK18, and caspase-3/-7 levels as well as tolerance of the drug, following larger phase II trials could not confirm protective effects on fibrosis and portal hypertension (Garcia-Tsao et al., 2020; Harrison et al., 2020). Emricasan is not the only caspase-inhibitor that had a proven effect in a preclinical setting, which could not be translated to disease improvement in clinical application. Table 4 gives an overview about different caspase inhibitors. It seems that in MASH, the inhibition of caspase-1 may direct cells to alternative mechanisms of cell death, abrogating any effects of the drug on liver fibrosis and hepatocyte ballooning. However, since caspases are among the orchestrators of inflammatory and non-inflammatory cell death, it is both valuable and worthwile to continue investigating suitable anti-caspase drug candidates.

**TABLE 4:** Overview of pyroptosis-targeting drugs for MASH.

Therapeutic agent	Group	Pyroptosis target	Status	Ref.
MCC950	NLRP3 inhibitor	NLRP3	Preclinical (mouse)	Mridha et al. (2017)
IFM-514	NLRP3 inhibitor	NLRP3	Preclinical (mouse)	Torres et al. (2021)
Obeticholic acid	Bile acid analog	NLRP3	Preclinical (mouse)	Neuschwander-Tetri et al. (2015), Yang et al. (2017), Huang et al. (2021b), Rinella et al. (2022), Younossi et al. (2022)
			Clinical (Phase III): NCT02548351, NCT01265498	
Liraglutide	GLP-1 receptor agonist	NLRP3	Preclinical (mouse)	Armstrong et al. (2016), Yu et al. (2019)
			Clinica (Phase II): NCT01237119	
Semaglutide	long-acting GLP-1 receptor agonist	NLRP3	Preclinical (mouse)	Wang et al. (2021a), Newsome et al. (2021), Mollerhoj et al. (2022)
			Clinical: NCT02970942 (Phase II), NCT04822181 (Phase III)	
Emricasan	Pan-caspase inhibitor	Caspase-1	Preclinical (mouse)	Barreyro et al. (2015), Shiffman et al. (2019), Garcia-Tsao et al. (2020), Harrison et al. (2020)
			Clinical (Phase III): NCT02686762, NCT02960204	
VX-166	Pan-caspase inhibitor	Caspase-1	Preclinical (mouse)	Witek et al. (2009)
GS-9450	Caspase inhibitor (1, 8, 9)	Caspase-1	Clinical (Phase II)	Ratziu et al. (2012)
Disulfiram	Aldehyde dehydrogenase inhibitor	GSDMD	Preclinical (mouse)	Lei et al. (2022)
			Clinical (phase I)	

GLP-1, glucagon-like peptide-1.

### 5.3 Gasdermins

Gasdermins being blocked and preventing membrane permeability was nicely shown in mechanistic *in vitro* experiments. Treatment of BMDMs with punicalagin, a complex polyphenolic compound, selectively blocked GSDMD-mediated membrane permeabilization while leaving caspase-1 activation and IL-1β processing unaffected (Martin-Sanchez et al., 2016). Up to now, no specific GSDMD or general GSDM inhibitor has been tested in clinical trials for MASLD/MASH treatment. However, there are several pleiotropic-acting drugs, shown to affect GSDMD signaling, currently under preclinical and clinical investigations.

Disulfiram, an FDA-approved drug for treating alcohol addiction, and its active metabolite, bis(diethyldithiocarbamate)-copper complex (CuET), were found to inhibit pore formation by specifically blocking GSDMD. Disulfiram still allows for IL-1β and GSDMD processing but abrogates pore formation, thereby preventing IL-1β release and pyroptosis (Hu et al., 2020). In a subsequent study, it was shown that CuET inhibits the maturation of both GSDMD and GSDME, inhibiting both GSDMD-dependent and -independent pyroptosis (but not apoptotic caspase-3-dependent GSDME activation) and IL-1β release. The author additionally showed that CuET effectively inhibited upstream NLRP3 activation (Wang et al., 2021b). In the setting of MASH, a protective effect of Disulfiram and its metabolites was shown in various preclinical models (Schwartz et al., 2013; Liu et al., 2018; Lei et al., 2022; Yamagishi et al., 2022). In the most recent study, the effects of Disulfiram on the microbiota in MASH were investigated, and the authors performed an additional self-controlled phase-1 clinical trial including 23 healthy volunteers who received 250 mg Disulfiram once a day for 7 days. Reportedly, the drug was tolerated well, and transferring the fecal microbiota into germ-free mice ameliorated MASH indicating that Disulfiram has many effects apart from inhibiting GSDMD pathways (Lei et al., 2022). Focusing more on the GSDMD-related inhibitory effects, Yamagishi et al. (2022) demonstrated that in Benz [a]anthracene-treated and HFD-fed mice, the administration of Disulfiram effectively inhibited the export of IL-1β and IL-33 from primary HSCs. Additionally, liver tumor formation was suppressed upon treatment, suggesting the importance of GSDMD pore formation and cytokine release from HSCs on tumor formation. Activated by canonical and non-canonical pyroptotic pathways, the inflammatory caspases-1, -4, -5, and -11 all play an important role by regulating the GSDMD activation and therefore are appealing targets in inhibiting the inflammasome-gasdermin axis. Necrosulfonamide (NSA), which is known to inhibit human mixed-lineage kinase domain-like pseudokinase (MLKL) in necroptosis, acts as a direct chemical GSDMD inhibitor. The inhibitor was tested *in vivo* in a mouse sepsis model with a lethal dose of LPS. Upfront administration of NSA increased median survival by 6 h and decreased levels of IL-1β and IL-6, suggesting that NSA could be a useful compound in other inflammatory diseases (Rathkey et al., 2018). Indeed, the treatment of isolated primary human hepatocytes from steatotic livers with NSA leads to a reduction of intracellular triglyceride content (Majdi et al., 2020). In the same study, RIPA-56, a specific inhibitor of RIPK1, which positively regulates MLKL, was tested *in vivo* and showed a protective effect in a murine HFD-diet MASH model by improving mitochondria function and reducing fibrosis. GSDMD activation by neutrophil elastase is crucial in NET formation, a special form of neutrophil cell death that releases chromatin structures to the extracellular space (Sollberger et al., 2018). Sollberger et al. (2018) used a compound (LDC7559) in their study, which potently inhibited GSDMD and thereby NETosis. LDC7559 was found to reduce IL-1β release and inhibit GSDMD membrane localization, suggesting that it affects GSDMD cleavage or membrane integration. The authors concluded that discovering LDC7559 as an inhibitor could be a starting point to target GSDMD function in a variety of diseases. However, until now, LDC7559 has only been proven to be effective in preclinical studies. All pyroptosis-targeting drugs that have been shown to have an effect in preclinical MASH models or clinical studies are summarized in Table 4.

## 6 Conclusion

New insights have enhanced our understanding of the mechanisms governing cell death, particularly shedding more light on the interplay between different types of cell death. In the context of MASLD and MASH, it is crucial to apply our knowledge of these pathways to better target the key cell types involved in MASH development. This can be accomplished by utilizing rodent models that are cell and liver specific. It is clear that we are only at the initial stages of comprehending how different cell types and their subsets contribute to aggravating or resolving MASH.

Upcoming studies will provide much needed insights into unanswered questions, including the role of the entire gasdermin family or negative regulators of pyroptosis in MASH. Although completely specific gasdermin inhibitors are still undergoing preclinical testing, drugs targeting multiple pathways, including pyroptosis, are already being examined in clinical studies. This underscores the significance of further exploring the diverse regulatory pathways of gasdermin-dependent cell death to identify new therapeutic targets for treating inflammatory diseases. Future studies will need to investigate the interactions between different cell death pathways and the cell-specific effects of inhibiting cell death effector proteins. The improved understanding of these intricate and complex processes will fuel advancements in drug development for targeting inflammatory disorders and MASH.
