# Corrigendum: Pyroptosis and gasdermins—Emerging insights and therapeutic opportunities in metabolic dysfunction-associated steatohepatitis

**DOI:** 10.3389/fcell.2024.1461581

**Published:** 2024-10-16

**Authors:** Christian Stoess, Aleksandra Leszczynska, Lin Kui, Ariel E. Feldstein

**Affiliations:** ^1^ Department of Pediatric Gastroenterology, University of California, San Diego, San Diego, CA, United States; ^2^ Department of Surgery, TUM School of Medicine, Klinikum rechts der Isar, Technical University of Munich, Munich, Germany

**Keywords:** pyroptosis, gasdermins, liver, steatotic liver disease, steatohepatitis, MASH, MASLD

In the published article, there was an error in the **Conflict of interest** statement-author, Ariel E. Feldstein was erroneously excluded. The corrected **Conflict of interest** statement appears below.

“Author AEF is an employee and stockholder of Novo Nordisk.

The remaining authors declare that the research was conducted in the absence of any commercial or financial relationships that could be construed as a potential conflict of interest.”

The author apologizes for this error and states that this does not change the scientific conclusions of the article in any way. The original article has been updated.

